# 

**Published:** 1891-12-05

**Authors:** 


PRICE ONE PENNY.

No. 271, Vol. XI.—December 5th, 1891.

[Registered at the General Post Office as a Newspaper for transmission in the United Kingdom and Abroad.]

WITH EXTRA NURSING SUPPLEMENT.
Per Annum, 6s. 6d., post free.
Monthly Parts, 6d.; post free, 8d.
Ditto per Annum, 8s., post free.

### THE HOSPITAL

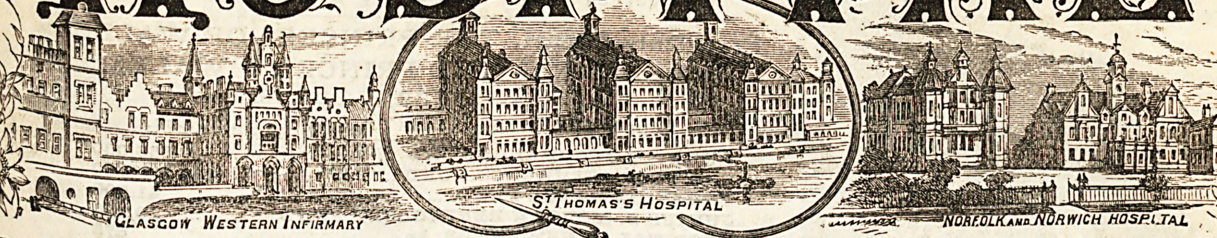

A WEEKLY JOURNAL OF
Science, Medicine, Nursing, and Philanthropy.

### SATURDAY, DECEMBER 5th, 1891.

**Feelings and Beliefs** - 109

**Passing Topics.**—Inside and Out - 110

**The Most Interesting Hospital in the World** - 111

**Some Scientific Aspects of Insanity.** V.—Causes of Insanity - 112

**Everybody's Page** - 113

**Notes and News** - 114

**General Charities—Scraps and Gleanings** - 115

**A Model Hospital** (*concluded*).—By Conrad W. Thies. - 119

**Annotations.**—The Bacillus of Typhoid Fever.—Leech's Life—Direct Representation: "Who Killed Cock Robin?" - 116

**The Practitioner's Mirror and Retrospect—**

Medicine.—On Cerebral Hæmorrhage in the Aged - 117

Surgery.—Thyroid Grafting in Myxœdema - 118

**Turning Over New Leaves.**—Two English Cardinals—Religious Tract Society's Publications - 119

**The Student of Medicine.**—Vacancies—Scholarships and Prizes—Events for the month of December.

### EXTRA SUPPLEMENT.—THE NURSING MIRROR.

**En Passant.**—The Nurse Dolls—The late Lord Lytton and his Nurse—Nursing in India—St. John's Home—Short Items—St Mary's Home - lv

**The Pen-Bron Hospital** - lvi

**The Victorian Exhibition** - lvii

**The Screen for the Princess of Wales** - lvii

**A Bed for a Sick Nurse** - lvii

**The Survival of the Fittest** - lvii

**Wants and Workers** - lvii

**For Reading to the Sick**—God's Hand in All Things - lvii

**A Nurse in Natal** (*continued*) - lviii

**Presentation** - lviii

**Everybody's Opinion.**—Her Majesty's Nursing Sisters—Ladies in Asylums - lix

**Appointment** - lix

**Notes and Queries** - lix

**Amusements and Relaxation** - lix

**Off Duty.**—"Dr. Sutters" (*concluded*) - lx

